# Prevalence of Various Systemic and Organ-Specific Autoimmune Markers in Addison’s Disease Patients Compared to Healthy Controls

**DOI:** 10.3390/jcm14113951

**Published:** 2025-06-03

**Authors:** Aylin Feyzullova, Georgi Kirilov, Atanaska Elenkova, Dobromir Tanev, Krassimir Kalinov, Sabina Zacharieva, Ralitsa Robeva

**Affiliations:** 1 Department of Endocrinology, Faculty of Medicine, Medical University-Sofia, 1000 Sofia, Bulgaria; aylin_feyzullova@abv.bg (A.F.); aelenkova@medfac.mu-sofia.bg (A.E.); 2 USHATE “Acad. Iv. Penchev”, 1000 Sofia, Bulgaria; drgkirilov@abv.bg (G.K.); zacharieva67@gmail.com (S.Z.); 3 Expert Center for Rare Endocrine Diseases, USHATE “Acad. Iv. Penchev”, 1000 Sofia, Bulgaria; 4 Department of Rheumatology, Clinic of Internal Medicine, National Multiprofile Transport Hospital “Tsar Boris III”, 1000 Sofia, Bulgaria; dobri_tanev@yahoo.com; 5 Scientific Affairs, Medical Center “Comac-Medical”, 1000 Sofia, Bulgaria; krassimir.kalinov@comac-medical.com

**Keywords:** Addison disease, lupus, rheumatoid arthritis, diabetes mellitus type 1, APS

## Abstract

**Background:** Addison’s disease (AD) is a rare disorder that often develops in the context of autoimmune polyglandular syndromes. However, the prevalence of rheumatological autoimmune diseases and corresponding autoimmune markers in AD is poorly investigated. Therefore, the present study aims to explore systemic and organ-specific immune markers in a cohort of AD patients from a single tertiary endocrine center. **Material and methods**: In total, 43 adult AD patients and 31 controls were included in the study. 21-hydroxylase autoantibodies (21OHAb), glutamic acid decarboxylase autoantibodies (GADAbs), zinc transporter-8 autoantibodies (ZnT8Abs), antibodies against nuclear antigens (ANAs), autoantibodies against cyclic citrullinated peptides (CCPAbs), rheumatoid factors (RFs), IgG autoantibodies against cardiolipin (ACLAbs), and autoantibodies against beta-2-Glycoprotein I (β2-GPIAbs) were measured in all participants. **Results**: An increased prevalence of antibodies against RFs (27.91% vs. 0%, *p* < 0.001) and ANAs (13.95% vs. 0%, *p* = 0.037) was found in AD patients compared to controls. Moreover, the titers of 21-hydroxylase and RF antibodies correlated positively (r = +0.269, *p* = 0.020). The AD patients tended to show an increased prevalence of subthreshold ACL antibody reactivity compared to controls. All patients diagnosed with type 1 diabetes mellitus were GADAb- but not ZnT8Ab-positive. **Conclusions**: The results show an increased prevalence of ANA and RF positivity in AD patients compared to controls and a significant association between 21-OHAb and RF positivity. ZnT8Ab positivity was not typical for adult AD patients from our ethnic group, while GADAbs were an essential marker for autoimmune diabetes mellitus. Extensive studies in different ethnic groups are needed to establish the clinical significance of various immunological markers for AD comorbidity and the appropriate follow-up protocols for patients with different antibody positivity.

## 1. Introduction

Addison’s disease (AD) is a rare disease with a prevalence of 82–117 per million in Central and South Europe and 140–221 per million in North Europe [1,2,3,4,5]. Nowadays, more than three-quarters of adult AD cases in the same region are caused by autoimmune adrenal impairment, while infectious, genetic, and other etiological factors are significantly rarer [6].

AD occurs as an isolated disease in less than 40% of patients, while in most affected individuals, it coexists with other autoimmune diseases [7,8]. In patients with autoimmune polyglandular syndrome type 1 (APSI), primary adrenal insufficiency (PAI) is associated with hypoparathyroidism, chronic candidiasis, and other autoimmune disturbances determined by pathological AIRE gene variants [9,10]. AD might also develop in the context of autoimmune polyglandular syndrome type 2 (APSII), including type 1 diabetes mellitus (T1DM) and autoimmune thyroid disorder (AITD) as main components [10,11]. Other autoimmune diseases may also affect APSII patients, e.g., autoimmune gastritis, celiac disease, vitiligo, and alopecia [10]. The genetic predisposition for APSII is associated to a large extent with specific human leukocyte antigen (HLA) DQ/DR haplotypes, but genetic variants in CTLA-4 and PTPN22 may also contribute to the immune alterations [12,13,14].

The associations between different autoimmune endocrine diseases have been studied in detail, while the data focusing on the interrelations between endocrine and rheumatological autoimmune disturbances are scarce. Rheumatoid arthritis (RA) and systemic lupus erythematosus (SLE) have been found in 10.8% and 5.4% of patients with multiple endocrine autoimmune diseases [15], while chronic arthritis has been described in only 2% of APSII patients [12,16]. Additionally, systemic connective tissue diseases have been rarely described in AD cohorts, and only a few case reports have been published in the literature [7,8,17,18]. However, a recent large UK study has reported a high co-occurrence rate of different connective tissue diseases (SLE, RA, etc.) with adrenal insufficiency, though various causes for hypocortisolism have not been specifically addressed [19].

Therefore, the present study aims to investigate systemic and organ-specific immune markers in a cohort of AD patients from a single endocrine center, exploring their association with the clinical manifestations of autoimmune diseases.

## 2. Materials and Methods

### 2.1. Study Participants

A total of 43 adults (18–75 years) diagnosed with AD and followed up in a single tertiary endocrine clinical center were included in the study. The diagnosis was based on current guidelines (decreased morning basal or Synacthen-stimulated cortisol levels with at least a doubling of ACTH levels) [20,21].

Two patients (4.7%) had autoimmune polyglandular syndrome type 1 (APSI); in one of the APSI patients, the diagnosis was genetically proven, while in the other, it was based on clinical characteristics. Four women (9.3%) suffered from “classic” APSII, including AD, T1DM, and AITD, while 26 AD patients (60.4%) had at least one concomitant autoimmune disease. Eleven patients suffered from isolated AD. Individuals with tuberculosis, adrenal malignancies, adrenoleukodystrophy, “triple A” syndrome, congenital adrenal hyperplasia, or other known causes for adrenal dysfunction beyond autoimmunity were excluded from the study. Additionally, thirty-one individuals of similar age (25–73 years) were recruited as a control group. Only clinically healthy men and women without symptoms of hypocortisolism or active corticosteroid treatment were included in the control group. Written informed consent prior to study procedures was obtained from all participants, and the study was approved by the Ethics Committee of the Medical University-Sofia.

### 2.2. Study Protocol and Laboratory Investigations

A detailed history focusing on concomitant autoimmune diseases and medications used was taken from all participants. Age, weight, height, and blood pressure were measured, and body mass index (BMI) was calculated. The mean corticosteroid and mineralocorticoid replacement doses in patients were also calculated. After an overnight fast, early morning blood samples from PAI patients and controls were taken. Blood count, biochemistry, and hormonal parameters were measured by an automatic analyzer COBAS INTEGRA 400 plus, Roche Diagnostics (Basel, Switzerland). Biosamples (blood serum) for antibody investigations were stored at −80°. In all patients, 21-hydroxylase autoantibodies (21OHAbs), glutamic acid decarboxylase autoantibodies (GADAbs), zinc transporter-8 autoantibodies (ZnT8Abs), antibodies against nuclear antigens (ANAs), autoantibodies against cyclic citrullinated peptides (CCPAbs), rheumatoid factors (RFs), IgG class autoantibodies against cardiolipin (ACLAbs), autoantibodies against beta-2-Glycoprotein I (β2-GPIAbs), and IgG autoantibodies to thyroid peroxidase (TPOAbs) were measured. Immunological markers were measured using an enzyme-linked immunosorbent assay (ELISA) or a radioimmunological assay (RIA). Cut-off values recommended by the manufacturer, as well as the percentage of detectable antibodies, were considered in the interpretation of the results.

### 2.3. Immunological Assays

21OHAbs were measured by an ElisaRSR^TM^ 21-OH Ab kit; the manufacturer’s cut-off for a positive result was 0.4 U/mL. An ElisaRSR^TM^ GADAb kit was used for the quantitative determination of GADAbs. The assay’s measuring range was 5–250 U/mL, with a cut-off of 5 U/mL for positivity. ZnT8Abs were measured by an ElisaRSR^TM^ ZnT8 Ab kit with the assay’s measuring range between 10 and 2000 U/mL and a cut-off of 15 U/mL for positivity. A Demeditec ANA Screen ELISA DE7030 kit was used to detect ANAs. The cut-off index for negative results was <1.0. A Demeditec CCP Ab ELISA DE7760 kit was used to quantitatively measure IgG autoantibodies against cyclic citrullinated peptides (CCPs), and ≥20 U/mL was considered a positive result. The functional sensitivity was 1 U/mL. A Demeditec Rheumatoid Factor Screen ELISA DE7660 was used to quantitatively measure RF antibodies (RFs) with a cut-off value set at 25 U/mL for positivity and a functional sensitivity of 1 U/mL. A Demeditec cardiolipin Ab IgG/IgM ELISA DE7300 kit was used to measure ACLAbs quantitatively. The assay’s measuring range was 0–120 U/mL, and the cut-off was 10 U/mL, with values below considered negative. The functional sensitivity was determined to be 1 U/mL. A Demeditec Beta2-Glycoprotein I Screen ELISA DE7270 kit was used to measure β2-GPIAb quantitatively. The assay has a measuring range of 0–90 U/mL, with a cut-off value of 10 U/mL for distinguishing between negative and positive results. Functional sensitivity was determined to be 0.5 U/mL. An Anti-TPO [I-125] RIA Kit (REF: RK-36CT) was utilized for the direct quantitative determination of autoantibodies to thyroid peroxidase (TPO). A 25 IU/mL cut-off was accepted for positive results per the manufacturer’s instructions.

### 2.4. Statistical Analysis

Descriptive statistics and frequency analysis were used to present categorical variables. Fisher’s exact chi-square test was used to test the hypotheses of no differences. Continuous variables were presented as arithmetic means and standard deviations (SD) or medians with interquartile ranges (ICRs). Differences between the two groups were calculated using a Student’s two independent sample *t*-test. Correlations were established by Pearson’s correlation. A significance level of 5% was used for decision-making.

The SAS^®^ package version 9.4 (SAS Institute Inc., SAS 9.4 Help and Documentation, Cary, NC, USA: SAS Institute Inc., 2015–2022) and MedCalc^®^ Statistical Software version 23.1.3 (MedCalc Software Ltd., Ostend, Belgium; https://www.medcalc.org; (accessed on 24 May 2025)) were used for the calculations and the presentation of the results.

## 3. Results

The main characteristics of the AD patients and controls are presented in Table 1. Both groups were of similar age and sex ratio, and no differences in BMI were established. The AD patients had increased ACTH levels compared to controls, as expected. The sodium levels were lower, while TSH and creatinine levels were higher in AD individuals compared to controls. Age at AD diagnosis was similar in male and female patients (38.02 ± 14.04 years in women vs. 42.62 ± 16.93 years in men, *p* = 0.425). The mean duration of the disease was 13.21 ± 12.80 years.

**Figure 1:**
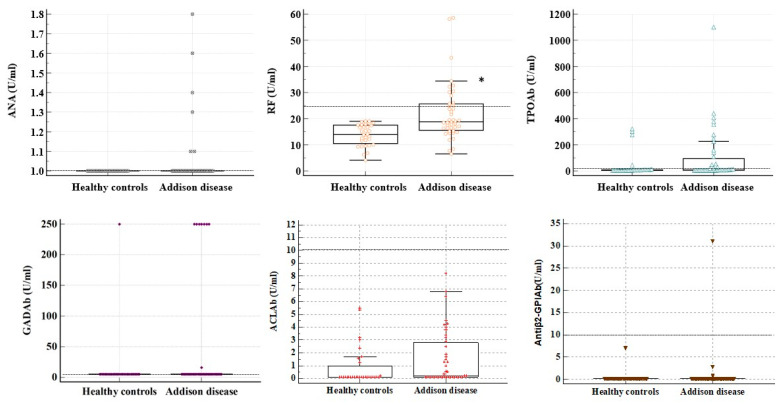
The quantitative distribution of antibodies in the investigated patients and controls. Legend: ANAs—IgG antinuclear antibodies; RFs—IgG, IgM, and IgA class rheumatoid factor antibodies; ACLAbs—IgG autoantibodies against cardiolipin; Antiβ2-GPIAbs—IgG, IgM, and IgA class autoantibodies against beta-2-Glycoprotein I; GADAbs—IgG glutamic acid decarboxylase autoantibodies; TPOAbs—IgG autoantibodies to thyroid peroxidase. The dotted line represents the positivity cut-off according to the manufacturer’s recommendations. *—*p* < 0.05.

The prevalence of autoimmune diseases and different antibodies is presented in Table 2 and Table 3 as well as in Figure 1.

**Figure 2:**
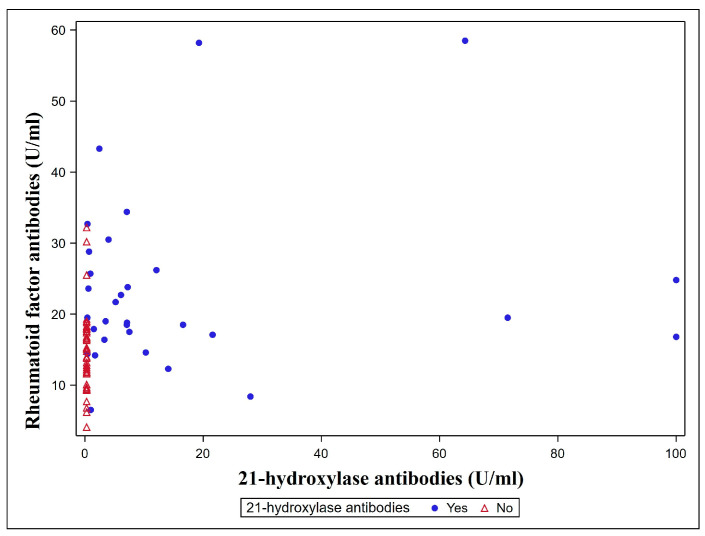
Distribution of rheumatoid factors (RF) antibodies and 21-OH autoantibodies.

The prevalence of 21-hydroxylase antibodies was specific for AD patients, and no positivity was found in the healthy controls (12.32 ± 24.61 IU/L vs. 0.3 U/L, *p* = 0.003). An increased prevalence of antibodies against RFs and ANAs was found in AD patients compared to the controls (Table 3). Moreover, the titers of 21-hydroxylase and RF antibodies were weakly positively related (r = +0.269, *p* = 0.020) (Figure 2). Conversely, no differences in the CCPAbs were found between the groups. None of the patients were previously diagnosed with systemic lupus erythematosus, while three patients had been diagnosed with rheumatoid arthritis (all CCPAb-negative and one RF-positive). Considering APSI patients, one had positive ANAs, and the other had positive RF antibodies, both without corresponding clinical symptoms.

None of the investigated women showed positive ACL antibodies, suggesting antiphospholipid syndrome, and only one of the AD patients was positive for antibodies against beta-2-Glycoprotein I (>10 U/L). This woman suffered from a polyglandular autoimmune syndrome, including autoimmune diabetes mellitus, vitiligo, and autoimmune thyroiditis, but had not reported any thrombotic incidences or pregnancy complications, suggesting antiphospholipid syndrome.

Nevertheless, the AD patients tended to show an increased prevalence of subthreshold ACL antibody reactivity compared to the healthy group, though the differences did not reach statistical significance (Table 3). Only three patients with AD had thrombotic incidents; thus, the associations between ACL-subthreshold antibody reactivity and thrombosis susceptibility could not be established. The prevalence of miscarriages in AD patients was 25.7% (9/35 women), and no differences in the miscarriage frequency were found between AD women with or without detectable ACL antibodies below the positivity threshold (27.8% vs. 23.5%, *p* = 1.000); however, in most patients, pregnancies had occurred before the AD diagnosis.

Four patients (9.3%) with AD but no individuals from the control group suffered from diabetes mellitus type 1. The prevalence of positive GADAbs was 18.60% in the AD group, with all diabetic type 1 patients being strongly positive (≥250 U/mL). Four AD patients and one healthy female were GADAb-positive without carbohydrate disturbances. Detectable ZnT8 antibodies were not found in our groups of patients and controls.

No differences in the various antibody positivity were established in AD patients based on the presence of 21-hydroxylase antibodies (*p* > 0.05 for all).

No differences in the immunological markers or electrolytes were found between patients using or not using fludrocortisone treatment (*p* > 0.05 for all).

## 4. Discussion

The present study focuses on the prevalence of some systemic and organ-specific autoimmune markers in patients with AD.

### 4.1. Autoimmune Markers for Systemic Connective Tissue Diseases

The results show a significantly increased prevalence of positive RFs and ANAs in patients compared to controls. Additionally, the RF titer has been positively related to 21-hydroxylase antibody levels. Approximately 7% of the AD patients had been previously diagnosed with RA, but most of them were negative for CCPAbs and RFs, which could be due to previous treatment or seronegative disease variants. The presence of RA is a rare finding in patients with AD, with only a few clinical cases described [22,23]. On the other hand, relative hypoadrenalism is a known feature of RA [24,25]. Steroidogenesis dysfunction in treatment-naïve RA patients has been associated with inadequate hypothalamic-pituitary stress response to chronic inflammation, but signs of primary adrenal impairment have also been described [26,27]. For instance, a low-dose ACTH stimulation test performed in RA patients shows a blunted cortisol response [28,29]. However, the associations between autoimmune markers in AD and RA have not yet been explored.

Interestingly, despite increased RF positivity, the investigated AD patients did not have pronounced complaints typical of RA. Lifelong corticosteroid replacement therapy is a necessity for AD patients [20,21]. Glucocorticoid treatment, currently recommended mainly as a “bridging” therapy, remains an important therapeutic approach for RA, even in the biological treatment era [30]. The mean age at AD onset in our cohort was 40 years, which precedes the peak incidence in RA at 50–54 years [31]. Therefore, we could hypothesize that corticosteroid treatment in AD patients might preclude the clinical manifestation of RA in some RF-positive AD patients. In support, in the described cases of patients with both diseases, the development of RA preceded the AD diagnosis [22]. On the other hand, muscular and joint pain are common symptoms of hypocortisolism [32], which could be resolved with corticosteroid treatments.

However, it should be emphasized that RF positivity is not specific to RA, and a myriad of systemic autoimmune, infectious, and oncologic diseases may be associated with an increased RF titer [33]. The highly specific antibodies for RA CCPAbs [34] were found six times less frequently than RF antibodies in our AD group, suggesting that the RF increase might be a non-specific sign of immunity alterations. Several genetic variations, e.g., PTPN22 and CTLA-4 polymorphisms [35,36,37], might predispose simultaneously to the development of AD, RA, and other systemic and organ-specific autoimmune diseases. However, it is unclear whether they could affect the antibodies production in AD patients. Thus, as a result, exploring these associations may be of clinical importance.

Almost 14% of our AD patients showed ANA positivity in contrast to controls who were ANA-negative. HLA-DR3 and HLA-DR4 haplotypes are common in SLE and APS type II individuals. Thus, they can predispose to increased antibody production [38]. On the other hand, SLE-related vasculitis might be an important factor compromising adrenal vascularization and function [39]. Nevertheless, the association of AD with SLE is a rare finding, with only about 20 cases described to date [38,39,40,41]. In only two cases, SLE developed in patients already diagnosed with AD [38,39,40,41]. Our AD patients did not show any SLE clinical characteristics, but a regular follow-up should be recommended.

Considering ACLAbs and Antiβ2-GPIAbs, only one of the patients (with APS type II but undetectable 21-hydroxylase antibodies) has been positive for antibodies against beta-2-Glycoprotein I. However, the woman has not developed clinically apparent antiphospholipid syndrome (APhS) yet. APhS is an important cause of thrombotic-associated adrenal injury [39,42]. Adrenal parenchymal bleeding might be found in the case of APhS because of the adrenal venous thrombosis increasing tissue vessel resistance and intracapsular pressure [43]. Studies have shown that 4 of 1000 European APhS patients suffer from AD [44], while adrenal failure affects up to 26% of patients with catastrophic APhS [45]. Thus, the presence of adrenal hemorrhage or microthrombosis has been included in the 2023 ACR/EULAR antiphospholipid syndrome classification criteria [46]. The association between APhS and autoimmune endocrine disorders might be mediated not only through vascular but also through autoimmune mechanisms. For instance, increased APhS antibodies without clinical implications have been described in patients with autoimmune thyroid disorders [47]. However, APhS is found in only one in 350 AD individuals in Italy [48] and is absent in our study. Nevertheless, the investigated AD patients tend to express an increased prevalence of subthreshold ACL antibody reactivity compared to healthy women. The patients have not shown any manifestations of subclinical antiphospholipid syndrome, but the possible effects on future thrombosis susceptibility need to be established. This is also of the utmost importance for pregnancy outcomes of young women with AD and detectable APhS antibodies.

### 4.2. Organ-Specific Autoimmune Markers

Approximately two-thirds of our AD patients had been diagnosed with an autoimmune thyroid disorder (AITD), though TPOAbs were positive in only half of them. L-thyroxin treatment has been associated with TPO decline in AITD patients [49], but long-lasting corticosteroid treatment may have an additional influence. The prevalence of AITD in Italian and Polish AD patients was higher, reaching 73–75% [16,50]. Conversely, in Scandinavian cohorts, it was slightly lower (48–59%) than in our group, though more than 60% of patients were TPOAb-positive [7,51].

The prevalence of diabetes mellitus type 1 (T1DM) in our group was 9.3%, but twice as many patients were GADAb-positive (18.6%). All T1DM patients were GADAb-positive, while other positive patients (and one positive control) had no carbohydrate disturbances. The prevalence of T1DM in our AD cohort was similar to the described prevalence in the UK (10%), Sweden (10.7%), and Poland (10%) [7,50,52]. The prevalence of GADAb positivity was also close to the reported data for Polish (20.0%), Norwegian (21%), and Swedish AD patients (23.2%) [7,8,50]. However, we did not find any patients positive for ZnT8Abs in our study, while in other countries, the prevalence of ZnT8Ab-positive AD patients varies between 8.5% and 19% [50,53]. According to a recently published study on 160 adult T1DM patients from the same ethnic group (with approximately 16 months of mean disease duration), positive ZnT8 antibodies have been found in only 53% of the investigated cohort [54]. However, GADAbs remain the most reliable immunologic marker, identifying over 90% of antibody-positive patients [54]. Thus, discrepancies in our study might be related to the small number of diabetic patients with AD, advanced age, and treatment with relatively high corticosteroid doses, as well as ethnic features. Notably, the long duration of T1DM in our AD group might also be a significant factor leading to decreased humoral auto-activity. After 25 years of disease duration, only 6.7% of DMT1 patients remain ZnT8Ab-positive, while a quarter of them are still GADAb-positive [55]. Thus, ZnT8 antibodies are a key T1DM marker in childhood and adolescence, but their prevalence decreases with age in the common T1DM population [56]. Therefore, the GAD antibodies might be the most reliable autoimmune markers for T1DM in adult AD patients, at least in our population. Additionally, strict glucose monitoring should be recommended for GAD-positive non-diabetic AD patients.

### 4.3. Treatment

The mean prednisolone dose of 6.88 mg in the investigated AD patients was above the recommended usual dose of 3 to 5 mg/daily [20], varying from 0 mg in a newly diagnosed patient to 10 mg/daily in patients based on their individual needs, concomitant conditions, and preferences as well as medical recommendations. Approximately 81% of patients were on fludrocortisone treatment based on their electrolytes, plasma renin activity, and blood pressure levels, as well as the opinion of the monitoring physicians. Unfortunately, some women had discontinued the prescribed fludrocortisone therapy at their discretion, which highlights the need for better patient education. However, the corticosteroid dose used by our patients did not differ from the doses used in several large AD studies (6.3–7 mg/d prednisolone equivalent) [7,16]. Similarly, in other studies, 83.6–89% of AD cohorts were on fludrocortisone treatment [7,50]. Nevertheless, the higher prednisolone dose used in our cohort could have influenced the patients’ immunological patterns and clinical characteristics. Corticosteroid use (16.8 mg prednisone daily) could decrease mean serum IgG by approximately 22% in asthmatic patients [57]. On the other hand, even physiological prednisolone doses below 3–5 mg might be effective for alleviating clinical complaints in rheumatoid arthritis [58]. Thus, the potential symptoms and immunological disturbances in AD patients might have been more pronounced on lower prednisolone doses. Nevertheless, more efforts should be taken for the close monitoring of glucocorticoid and mineralocorticoid replacement therapy in AD patients to avoid overdosing as well as undertreatment.

## 5. Conclusions

To the best of our knowledge, our study is the first to show the prevalence of multiple autoimmune markers for systemic connective tissue diseases in AD and their associations with AD-specific 21-hydroxylase antibodies [59]. The results show an increased prevalence of ANA and RF positivity in patients compared to controls and an association between 21-OHAb and RF positivity. ACLAb and ZnT8Ab positivity is not typical for AD patients from our ethnic group, while GADAbs are an essential marker for autoimmune diabetes. The study’s main limitations are the relatively small group of AD individuals and the lack of ANA-profile antibody investigations. The inclusion of only 43 patients limits the statistical power, particularly for detecting associations involving less prevalent autoimmune markers such as CCPAbs and Anti-β2GPIAbs. On the other hand, the lack of specific ANA subtype investigations may have led to the underdiagnosis of some subclinical SLE cases in our cohort as well as concomitant autoimmune disturbances, e.g., Sjögren’s syndrome. Additionally, 12 of our AD patients were 21-hydroxylase antibody-negative, which could be due to corticosteroid treatment and long disease duration but also other unknown non-immunological causes for the adrenal damage.

Nevertheless, reporting data from the understudied Eastern European populations might shed additional light on co-autoimmunity in the rare Addison’s disease. More extensive multiethnic studies are needed to reveal the clinical significance of immunological markers in AD and improve the surveillance protocols for comorbidities in the affected patients.

## Figures and Tables

**Table 1:** Main characteristics of the investigated groups. Data are presented as mean ± SD or as percentages.

Parameter	Healthy Controls *n* = 31	Addison Disease *n* = 43	*p*-Value
Age (years)	46.61 ± 12.91	52.09 ± 12.76	0.075
Sex (number of females; %)	23 (74%)	35 (81%)	0.461
BMI (kg/m^2^)	24.61 ± 3.71	24.14 ± 4.48	0.626
Systolic blood (mmHg)	111.8 ± 15.80	112.2 ± 14.21	0.905
Diastolic BP (mmHg)	71.06 ± 6.91	73.70 ± 9.49	0.171
Blood glucose (mmol/L)	5.33 ± 0.44	5.46 ± 1.39	0.578
K (mmol/L)	4.74 ± 0.40	4.81 ± 0.88	0.660
Na (mmol/L)	146.5 ± 2.02	142.1 ± 7.68	<0.001
Creatinine (µmol/L)	68.27 ± 13.06	75.74 ± 15.75	0.029
ACTH (pmol/L)	3.66 ± 1.52	140.79 ± 173.10	<0.001
TSH (µIU/mL)	2.06 ± 0.98	5.08 ± 7.75	0.017
Mean prednisolone dose (mg) *	-	6.88 ± 2.17	NA
Fludrocortisone therapy (%)	-	81.4%	NA

Legend: BMI—body mass index; TSH—thyroid-stimulating hormone; ACTH—adrenocorticotropic hormone; NA—not applicable. * Hydrocortisone is not available in the country; therefore, most patients are on prednisolone treatment; doses used vary between 0 (in a newly diagnosed patient) and 10 mg per day.

**Table 2:** Prevalence of different autoimmune diseases in Addison disease patients compared to controls. Data are presented as numbers (percentages).

Autoimmune Disease	Healthy Controls *n* = 31	Addison Disease *n* = 43
Type 1 diabetes mellitus	0 (0%)	4 (9.30%)
Autoimmune thyroid disease	6 (19.35%)	29 (67.44%)
Vitiligo	0 (0%)	5 (11.63%)
Alopecia	0 (0%)	3 (6.98%)
Hypoparathyroidism	0 (0%)	1 (2.32%)
Pernicious anemia	0 (0%)	2 (4.65%)
Rheumatoid arthritis	0 (0%)	3 (6.98%)

**Table 3:** Prevalence of different antibody positivity in Addison disease patients compared to controls. Data are presented as numbers (percentages).

Parameter	Healthy Controls *n* = 31	Addison Disease *n* = 43	*p*-Value
21OHAbs (*n*, %)	0 (0%)	31 (72.09%)	<0.001
CCPAbs (*n*, %)	0 (0%)	2 (4.65%)	0.506
ANAs (*n*, %)	0 (0%)	6 (13.95%)	0.037
RFs (*n*, %)	0 (0%)	12 (27.91%)	<0.001
ACLAbs (*n*, %) *	9 (29.03%)	23 (53.49%)	0.056
Antiβ2-GPIAbs (*n*, %) *	1 (3.23%)	3 (6.98%)	0.635
GADAbs (*n*, %)	1 (3.23%)	8 (18.60%)	0.070
ZnT8Abs (*n*, %)	0 (0%)	0 (0%)	-
TPOAbs (*n*, %)	(4) 12.9%	(14) 32.56%	0.060

*—percentage of subthreshold antibody reactivity. Legend: 21OHAbs—IgG antibodies against 21-hydroxylase; CCPAbs—IgG autoantibodies against cyclic citrullinated peptides; ANAs—IgG antinuclear antibodies; RFs—IgG, IgM, and IgA class rheumatoid factor antibodies; ACLAbs—IgG autoantibodies against cardiolipin; Antiβ2-GPIAbs—IgG, IgM, and IgA class autoantibodies against beta-2-Glycoprotein I; GADAbs—IgG glutamic acid decarboxylase autoantibodies; ZnT8Abs—zinc transporter 8 autoantibodies; TPOAbs—IgG autoantibodies to thyroid peroxidase.

## Data Availability

Data are available from the corresponding author after reasonable request and with permission from the local authorities.
